# Hazelnut extract-loaded nanostructured lipid carriers and evaluation of their antioxidant properties

**DOI:** 10.3389/fbioe.2022.953867

**Published:** 2022-08-05

**Authors:** Melis Emanet, Özlem Şen, Francesca Pignatelli, Chiara Lavarello, Andrea Petretto, Gianni Ciofani

**Affiliations:** ^1^ Istituto Italiano di Tecnologia, Smart Bio-Interfaces, Pontedera, Italy; ^2^ IRCCS Istituto Giannina Gaslini, Core Facilities-Clinical Proteomics and Metabolomics, Genova, Italy

**Keywords:** hazelnut extract, nanostructured lipid carriers, choline, natural materials, antioxidant agents

## Abstract

Reactive oxygen species (ROS) are a common hallmark of many degenerative diseases, developing in all those cases where a failure of physiological antioxidant mechanisms occurs (in particular, antioxidant enzymes and the glutathione system), or in case of exposure to an extremely high level of oxidants. In this regard, antioxidant natural extracts are promising compounds as preventive or therapeutic agents against ROS-dependent degenerations. In this study, a deep investigation of hazelnut (*Corylus avellana*) extract has been performed in terms of mass spectroscopy, evaluation of phenolic content, and antioxidant capacity. Then, nanostructured lipid carriers (NLCs) have been exploited for encapsulation of the hazelnut extracts in order to achieve prolonged bioactivity, increased stability, and targeting through a sustainable delivery approach. The hazelnut extract-loaded NLCs (NE_NLCs) have been deeply characterized for their stability, production yield, and encapsulation efficiency. Moreover, NE_NLCs showed optimal cytocompatibility on human dermal fibroblast (HDF) cells, as well as excellent antioxidant activity, upon pro-oxidant stimulus on HDF cells.

## 1 Introduction

In nature, plenty of products contain biologically active compounds that own antioxidant, anti-inflammatory, and anti-microbial potentials, thus showing therapeutic and protective effects against oxidative stress-related aging processes and degenerative diseases (Fadaka et al., 2019). Living organisms possess a variety of antioxidant mechanisms to scavenge over-produced ROS in healthy physiological conditions; however, in case of high levels of oxidant accumulation in the tissues, or of exposure to an excessive rate of oxidants in pathological conditions (Zhang et al., 2011), endogenous antioxidants may not be enough, and a continuous exogenous antioxidant income is required, for example from natural sources like fruits, nuts, spices, vegetables, and mushrooms (Xu et al., 2017; Morgun et al., 2021).

Extensive investigations of natural materials indicated their rich content in vitamins, phenolic groups, essential oils, hydrocolloids, proteins, fibers, polysaccharides, and other organic compounds that make them suitable as excellent nutraceutical agents (Oliveira et al., 2007). In this regard, phenols are mainly composed of flavonoids -including anthocyanins, flavonols, flavones-, and of non-flavonoids -including phenolic acids, lignins, stilbenes- (Kornsteiner et al., 2006); all these compounds play a key role in reactive oxygen species/reactive nitrogen species (ROS/RNS) scavenging activity, as strong antioxidant agents. The phenol-dependent reduction of oxidative stress also inhibits the potential inflammatory reactions systematically activated in the organisms in case of degenerations (Bernardo-Colon et al., 2018). Moreover, a major task of phenols in plants regards their defense role against microorganisms: they in fact penetrate through the bacterial membrane or the cell wall altering membrane fluidity and cell wall integrity, thus resulting in microorganisms’ death (Tariq et al., 2019).

Given the excellent benefits of these natural materials, great efforts are focused on the obtainment of high amounts of target-specific active compounds, in particular considering the development of specific extraction techniques for each material/component, such as herbs, nuts, fruits together with their skin, seed, shell, and leaves (Selli et al., 2022). This need became particular urgent concerning hazelnuts: with their rich nutrient content, they play a major role in the human diet and health owing to a high content (60% in weight) of essential fatty acids (in particular oleic acid) as well as of other components such as proteins, carbohydrates, dietary fibers, phenols, vitamin E, minerals, phytosterols, and squalene (Alasalvar and Bolling, 2015; Pelvan Pelitli et al., 2017).

In the last 5 years, over 960 thousand hectares in the world are dedicated to hazelnut production; Turkey, with 610 thousand tons of hazelnuts in a year, is the world’s largest producer, accounting for the 62% of the global production. Considering this huge production, many researchers started to investigate Turkey’s nut potential, by evaluating extracts from skin/hull, kennel, and leaves (Lainas et al., 2016). Recent reports from the food and drug administration (FDA) indicate that, as a rich monounsaturated fatty acid (MUFA) source, hazelnuts tend to raise high-density lipoprotein (HDL) cholesterol blood levels, while lowering low-density lipoprotein (LDL) cholesterol and triglycerides, thus providing good protection against cardiovascular diseases. In the literature, phenols extracted from hazelnuts show great qualitative and quantitative diversity considering the cultivation region and the extraction methods. Senter *et al.* identified eight phenolic acids, including p-hydroxybenzoic acid, p-hydroxyphenylacetic acid, vanillic acid, protocatechuic acid, syringic acid, gallic acid, caffeic acid, and ferulic acid from nine different hazelnuts produced in the United States (Senter et al., 1983). Yurttaş *et al.* isolated and tentatively identified six phenols (p-hydroxybenzoic acid, epicathechin and/or caffeic acid, sinapic acid, and quercetin) in the hazelnuts produced in Turkey and United States (Yurttas et al., 2000). Amaral *et al.* identified and quantified further four phenols, namely 3-caffeoylquinic acid, 5- caffeoylquinic acid, caffeoyl tartaric acid, and coumaroyl tartaric acid in the hazelnuts cultured in Portugal (Amaral et al., 2005).

Historically, the hazelnut leaves were widely used in traditional medicine to treat hemorrhoids, varicose veins, phlebitis, and lower limbs edema, because of their astringent, vasoprotective, and anti-edema properties. Recent studies indicated that the hazelnut leaves are also a rich source of phenols, in particular 3-, 4- and 5-caffeoylquinic acids, caffeoyltartaric acid, p-coumaroyltartaric acid, myricetin-rhamnoside, quercetin 3-rhamnoside, and kaempferol 3-rhamnoside (Oliveira et al., 2007).

Considering extraction methods, the solvent-based one is a commonly exploited technique that presents high production capacity, low energy consumption, easy operation, and cost- and time-efficiency (Xu et al., 2017). Ethanol and methanol are often used for their extraction capacity of wide variety of active materials at high extent (Marino et al., 2021; Vergun et al., 2022). Acetone and dimethylsulfoxide (DMSO) have been also exploited for the extraction of organic compounds (Sricharoen et al., 2015): in general, the polarity of the solvent is strictly related to the resulting extracted molecules (Widyawati et al., 2014). In order to improve the extraction efficiency, the diffusion of the active compounds form the solid matrix into the solvent can be facilitated by application of microwave (Bener et al., 2022) or by physical/mechanical aids such as maceration and ultrasound application (Di Michele et al., 2021).

Aiming at elaborating a delivery strategy for hazelnut extracts, lipid-based nanosystems are particularly appropriate for the loading of the compounds, providing protection and stabilization in physiological media as well as high diffusion capacity through the cell membrane (Puri et al., 2009). Liposomes, solid lipid nanoparticles (SLNs), and nanostructured lipid carriers (NLCs) are developed for efficient drug encapsulation and drug accumulation in the target tissue (Abbott, 2013), by providing drug stability and controlled release of the therapeutic molecules (Tapeinos et al., 2017). Among lipid-based nanovectors, NLCs have been developed during the last decade as advantageous tools with respect to more traditional liposomes and SLNs, since they present both solid and liquid lipids at physiological temperatures that provide a high level of flexibility, drug loading, and excellent biocompatibility and biodegradability (Dolatabadi et al., 2021). In particular, the high flexibility of the NLCs avoids drug leakage and improves nanovector diffusion capacity through the cell membrane (López-García and Ganem-Rondero, 2015), all features that make them suitable also for the delivery of antioxidant agents (Ahmadzadeh et al., 2020), including hazelnut extracts. In the literature, NLCs have been proposed for the delivery of strong antioxidants such as resveratrol (Sen et al., 2021), curcuminoids (Dolatabadi et al., 2021), idebenone (Martinelli et al., 2020), and linoleic acid (Sadat et al., 2020).

In the present study, hazelnut extraction has been performed in hydroethanolic solution, and the product has been analyzed in terms of mass spectroscopy, total antioxidant capacity, and phenolic content. Then, following the development of NE_NLCs, the nanostructure stability and encapsulation efficiency have been extensively investigated, and dose-dependent cytocompatibility and cellular uptake have been evaluated on human dermal fibroblasts (HDFs). Eventually, NE_NLCs have been tested as oxidative stress-protective agents on *tert*-butylhydroperoxide (tBH)-stimulated HDFs.

## 2 Materials and methods

### 2.1 Extraction of hazelnut active compounds

In this study, the pesticide-free and bioactive ingredient-rich Turkish hazelnuts named “fatty” were obtained from fields located in Sakarya, in the northwest of the country. In the preparation of the hazelnuts for extraction, fruits have been delicately minced and de-hydrated through freeze-drying, in order to minimize water content, before obtaining a powder to optimize the extraction yield. The extraction has been performed in hydroethanolic solution, the latter owning polarity similar to that one of most of the active substances of interest (Rodríguez-Ruiz et al., 2022). The process of hazelnut extraction comprises the following steps: 1) solvent penetration through the natural material, 2) solubilization of the active ingredients, and 3) diffusion of the solutes in the liquid phase. The active ingredient-rich extract has been separated from solid residues through several gentle centrifugation steps.

For the extraction of active substances, hazelnuts were separated from the shell and finely minced in powder by a blade mixer. Then, the hazelnut powders were freeze-dried using a Freeze Dryer (FreeZone Freeze Dryer by Labconco) for the removal of water in order to maximize the extraction of active compounds. For this purpose, the hazelnut powder was frozen at -80°C for 24 h, and then freeze-dried for 12 h. To perform the extraction, 6 g of freeze-dried hazelnut powder was dissolved in 40 ml of ethanol solution in water (1:1 v/v), and continuously stirred at 150 rev/min on a shaker at 65°C for 20 min. Then, the extract mixture was filtered by using Whatman Grade 1 filter paper to eliminate solid residues. Further purification was performed three times by centrifugation at 5,000 rpm for 15 min. The resulting supernatant was collected and stored at -20°C in dark for future characterization and experiments.

In order to assess the obtained extract concentration, 1 ml of extract has been freeze-dried and the resulted dried mass weighed.

### 2.2 Characterization of hazelnut extracts

#### 2.2.1 Mass spectroscopy analysis

The molecular composition of the extracts was analyzed by using a Vanquish Horizon UHPLC coupled to Q-Exactive Orbitrap mass spectrometer. The extracted molecules were diluted (1:10 dilution) in methanol and 5 μL samples were directly injected into the reverse phase (RP) column. The molecular separation was carried out at 40°C with an ACQUITY C18 BEH 1.7 μm 2.1 × 100 mm column (Waters S.p.A.). The linear gradient started from 1% B phase (acetonitrile, 0.1% formic acid) and 99% A (H_2_O, 0.1% formic acid) to 100% B in 15 min with a 250 μL/min flow rate, then the columns were stabilized for 5 min with 1% phase B. The experiments were performed in data-dependent acquisition mode alternating full MS and MS/MS scans. The precursors were ionized using an electrospray at +3.9 kV and the inlet capillary temperature was held at 300°C. Nitrogen sheath gas and nitrogen auxiliary gas were set at a flow rate of 35 and 5 arbitrary units (AU), respectively. Single MS survey scans were performed in the Orbitrap, recording a mass window between 70 and 1,000 m/z with automatic gain control (AGC) target of 10^6^, at the maximum injection time of 100 ms and a resolution of 35,000 at 200 m/z. Data-dependent MS/MS analysis was performed in top speed mode with a 2 s cycle-time with an isolation window of 1.2 m/z and an exclusion list for 2 s. The intensity threshold was set at 1.6∙10^5^ using an isolation window of 1.4 Da 17,500 resolution, 10^5^ AgC, and 50 ms maximum injection time were used for the MS2 scan. Raw data files were processed by Compound Discoverer™ 3.1 software. Briefly, raw files were aligned with an adaptive curve setting with 5 ppm mass tolerance and a 0.8 min retention time shift. Unknown compounds were detected with a 5 ppm mass tolerance, 3 signal to noise ratio, 30% of relative intensity tolerance for isotope search, 300,000 minimum peak intensity, and then grouped with 5 ppm mass and 0.2 min retention time tolerances. A procedural blank sample was used for background subtraction. Molecules were identified by using the mzCloud spectral library. Only the best match higher than 85 was considered.

#### 2.2.2 Total antioxidant capacity, total phenolic group assessment, and choline quantification

The total antioxidant capacity of the hazelnut extract has been analyzed by a total antioxidant capacity detection kit (Sigma-Aldrich) following the manufacturer’s instructions. 50 µL of hazelnut extract, or 50 µL of Trolox solution at increasing concentrations (0, 80, 120, 160, 200, and 400 µM), have been mixed with 100 µL of a Cu^2+^–containing solution (100 µL) and incubated at room temperature in the dark for 90 min. The absorbance of the samples was thereafter assessed at 570 nm by using a multi-plate reader (Victor3, PerkinElmer), and the Trolox-equivalent antioxidant capacity of hazelnut extract was calculated according to the obtained standard curve.

Total phenolic group evaluation in the hazelnut extract has been performed by using the Folin Ciocalteu reagent (Sigma-Aldrich), which shows the total phenolic group content in a sample with respect to a standard compound (in this case, tannic acid at increasing concentrations: 0, 6.25, 12.5, 25, and 50 μg/ml). The assessment has been performed in 24 well-plates by adding 1,580 µL of dH_2_O, 20 µL of extract (600 μg/ml) or tannic acid, 100 µL of Folin Ciocalteu reagent, and 300 µL of sodium carbonate (20% w/v in water). After mixing, the plates have been incubated at 37°C for 35 min. Then, the absorbance of the samples was assessed at 800 nm by using the plate reader, and the tannic acid-equivalent phenolic content of the hazelnut extract was calculated according to the obtained standard curve.

The concentration of choline in the extracts was assessed by a total choline/acetylcholine quantitation assay (Sigma-Aldrich MAK056) by following the manufacturer’s instructions. For the preparation of the assay, all the reagents were warmed to room temperature. For the analysis of choline content in the sample, 50 mM of choline standard were diluted in buffer solution at increasing concentrations (0, 0.02, 0.04, 0.06, 0.08, and 0.1 mM); for quantification, 10 µL of hazelnut extract were diluted in 40 µL of buffer solution. Thereafter, 2 µL of choline enzyme mix and 2 µL of probe solutions were added and incubated at room temperature in the dark for 30 min. The absorbance has been eventually assessed at 570 nm using the plate reader. The choline content of hazelnut extract was calculated according to the performed measurements and to the obtained standard curve.

### 2.3 Synthesis of NLCs and NE_NLCs

The NLC fabrication has been performed by following a previously optimized method developed by our group (Sen et al., 2021). The NLCs were prepared by hot homogenization technique using glyceryl dibehenate (4.5, w/v) as solid lipid and oleic acid (0.5, w/v) as liquid lipid, jointly to a colloidal stabilizer, 1,2-distearoyl-*sn*-glycero-3-phosphoethanolamine-poly (ethylene glycol) (DSPE-PEG, 0.05%, w/v), and surfactant (poloxamer 188, 0.2%, w/v). Briefly, glyceryl dibehenate was weighted in a glass bottle, and oleic acid, poloxamer 188, and DSPE-PEG were placed in another vial and heated at 75°C; thereafter, the two phases were mixed and incubated for 10 min at 75°C before a sonication procedure (FisherbrandTM Q125 Tip Sonicator) for 15 min at 90% amplitude of power. NE_NLCs have been prepared analogously, but adding 2.4 mg/ml of extracts to the mixture (4.5% w/v with respect to glyceryl dibehenate). At the end of the procedure, the samples were stored at 4°C for at least 1 h. Obtained NLCs and NE_NLCs were filtered first with a 0.45 μm filter to eliminate big aggregates and then with a 0.2 μm filter (Sartorius Minisart Plus Syringe Filters) for further cleaning; a final purification with an Amicon^®^ Ultra-4 centrifugal filter (100 kDa, Sigma-Aldrich) at 5,000 rpm for 5 min was performed, three times, to remove non-loaded extracts. The samples were stored in glass vials in dark at 4°C for further experiments.

For fluorescence imaging (please see in the following for details), NLCs have been labeled with Vybrant™ DiO dye (Invitrogen). Briefly, 500 μg/ml of NLCs were mixed with 5 µM of the dye and continuously shaken at a 150 rev/min for 16 h at room temperature. Eventually, to remove free DiO dye, a further purification step was performed with an Amicon^®^ Ultra-4 centrifugal filter (2000 rpm for 5 min).

### 2.4 Characterization of NLCs and NE_NLCs

Transmission electron microscopy (TEM) images of NLCs and NE_NLCs have been acquired by using a JEOL JEM1011 transmission electron microscope equipped with a thermionic electron source (tungsten) and operating at 100 kV. Samples were diluted to 1 mg/ml and a drop of the dispersion was placed on a Cu grid, coated with ultrathin amorphous carbon film, previously plasma-treated (O_2_+Ar plasma, 10 W, 2 min) to remove hydrocarbon residues from carbon film deposition. To enhance the contrast of lipid particles, the procedure includes a 60 s negative staining by using uranyl acetate solution (1% v/v).

The colloidal stability of NLCs and NE_NLCs was evaluated by measuring their size distribution and ζ-potential using a ZetaSizer (Malvern Instruments). 60 μg/ml of samples were prepared in dH_2_O at a final volume of 1 ml and measured three times at room temperature.

The production yield of NLCs and NE_NLCs was assessed by measuring the dry weight of the samples upon freeze-drying (FreeZone Freeze Dryer by Labconco), according to Equation 1.

$$\text{Production}\;\text{Yield}\left(\text{\%}\right)=\left(\frac{Wtheoretical-Wfreeze\_dried}{Wtheoretical}\right)\cdot 100$$ (1)

For the evaluation of the extract encapsulation efficiency, upon freeze-drying NE_NLCs have been dissolved in methanol/water (1:1 v/v) solution and heated at 75°C for 2 h under stirring. Then, the total choline assay has been exploited to indirectly assess the extract content in the nanoparticles.

The total antioxidant capacity of NLCs and NE_NLCs was analyzed as described in the previous section for the plain extract, by using 0.02, 0.03, 0.06, 0.13, 0.25, 0.50, and 1.00 mg/ml of either NLCs or NE_NLCs. The antioxidant power was again expressed in terms of Trolox equivalent.

### 2.5 Cellular studies

#### 2.5.1 Cell culture

Human dermal fibroblasts (HDFs) were cultured in Dulbecco’s Modified Eagle’s Medium supplemented with 10% fetal bovine serum (FBS, Gibco), 1 mM L-glutamine (Gibco), and 100 IU/ml of penicillin-streptomycin (Gibco). The cells were incubated at 37°C under 5% CO_2_ atmosphere. The experiments were performed on HDFs within 10–15 passages.

#### 2.5.2 Cytocompatibility analysis of NLCs and NE_NLCs

Metabolic activity in cells was assessed using WST-1 colorimetric assay (BioVision), performed by following the manufacturer’s instructions. The cells have been seeded at a density of 1∙10^4^ cells/cm^2^ in 96 well-plate and incubated overnight. Then, cultures have been treated with NLCs and NE_NLCs at increasing concentrations (0, 5, 10, 25, 50, 100, and 250 μg/ml) and further incubated for 24 h. At the endpoint, the medium has been replaced with WST-1 reagent (10% v/v in cell medium), and cells were incubated for 45 min. Eventually, absorbance measurement at 440 nm has been performed by using the Perkin Elmer Victor X3 microplate reader; experiments have been performed in triplicate.

Cell proliferation upon incubation with NLCs and NE_NLCs (same treatment described for metabolic activity assessment, but with cell cultures performed in 24-well plates) has been analyzed by using the Quant-iT PicoGreen dsDNA assay kit (Invitrogen) by following the manufacturer’s instructions. At the endpoint, cells were rinsed and left under 500 µL of Milli-Q before three freezing/thawing cycles between -80°C and room temperature, to allow for complete cell lysis. After a centrifugation step to remove cellular debris, the dsDNA content has been evaluated by mixing 100 µL of reaction buffer, 50 µL of cell lysate, and 150 µL of PicoGreen reagent. After a 10 min incubation under shaking at room temperature, fluorescence emission (directly proportional to the dsDNA content and thus to cell number), has been measured by using the Perkin Elmer Victor X3 microplate reader (*λ*_*ex*_ = 485 nm; *λ*_*em*_ = 535 nm); experiments have been performed in triplicate.

#### 2.5.3 Cellular internalization

For confocal microscopy imaging, HDFs were seeded at 1∙10^4^ cells/cm^2^ in µ-Dishes (35 mm, Ibidi). After 24 h of incubation, 100 μg/ml of DiO-labelled NE_NLCs were added and incubated for a further 24 h. Then, the cells were fixed with 4% paraformaldehyde (PFA, Sigma-Aldrich) at 4°C for 20 min, and rinsed three times with PBS (Sigma-Aldrich). Thereafter, cells were treated with Hoechst (1:1,000 v/v, Invitrogen) and TRITC-phalloidin (1:200 v/v, Sigma-Aldrich) at 37°C for 45 min for nuclei and *f*-actin staining, respectively. Finally, 2D images and 3D rendering have been acquired with a confocal fluorescence microscope (C2 system Nikon).

For flow cytometry analysis, HDFs (1∙10^4^ cells/cm^2^) were seeded in a 24-well plate and incubated for 24 h. Then, they were treated with 100 μg/ml of DiO-labelled NE_NLCs. After a further 24 h of incubation, the cells were washed and centrifuged at 610 *g* for 5 min. The pellets were resuspended in PBS and analyzed by flow cytometry (Beckman Coulter CytoFLEX; *λ*_*ex*_ = 488 nm; *λ*_*em*_ = 530 nm).

#### 2.5.4 ROS detection

NE_NLC protective effects against ROS have been tested in the presence of *tert*-butyl hydroperoxide (tBH, Sigma-Aldrich) solution, as a pro-oxidant insult. HDFs were seeded at 1∙10^4^ cells/cm^2^ and incubated for 24 h at 37°C. Thereafter, they have been treated with 200 µM tBH and Nut Ext (70 μg/ml), NLCs (100 μg/ml), or NE_NLCs (100 μg/ml). The experiments have been performed on both insulted (200 µM tBH) and non-insulted (0 µM tBH) cultures. After 24 h of incubation, the cells were washed with PBS, collected using trypsin, and centrifuged at 1,000 *g* for 7 min. HDFs have been stained using 5 µM CellROX™ Green Reagent (Invitrogen) in PBS for 30 min at 37°C and analyzed by flow cytometry (Beckman Coulter CytoFLEX; *λ*_*ex*_ = 498 nm; *λ*_*em*_ = 522 nm).

## 3 Results and discussion

### 3.1 Production and characterization of hazelnut extract

**FIGURE 1:**
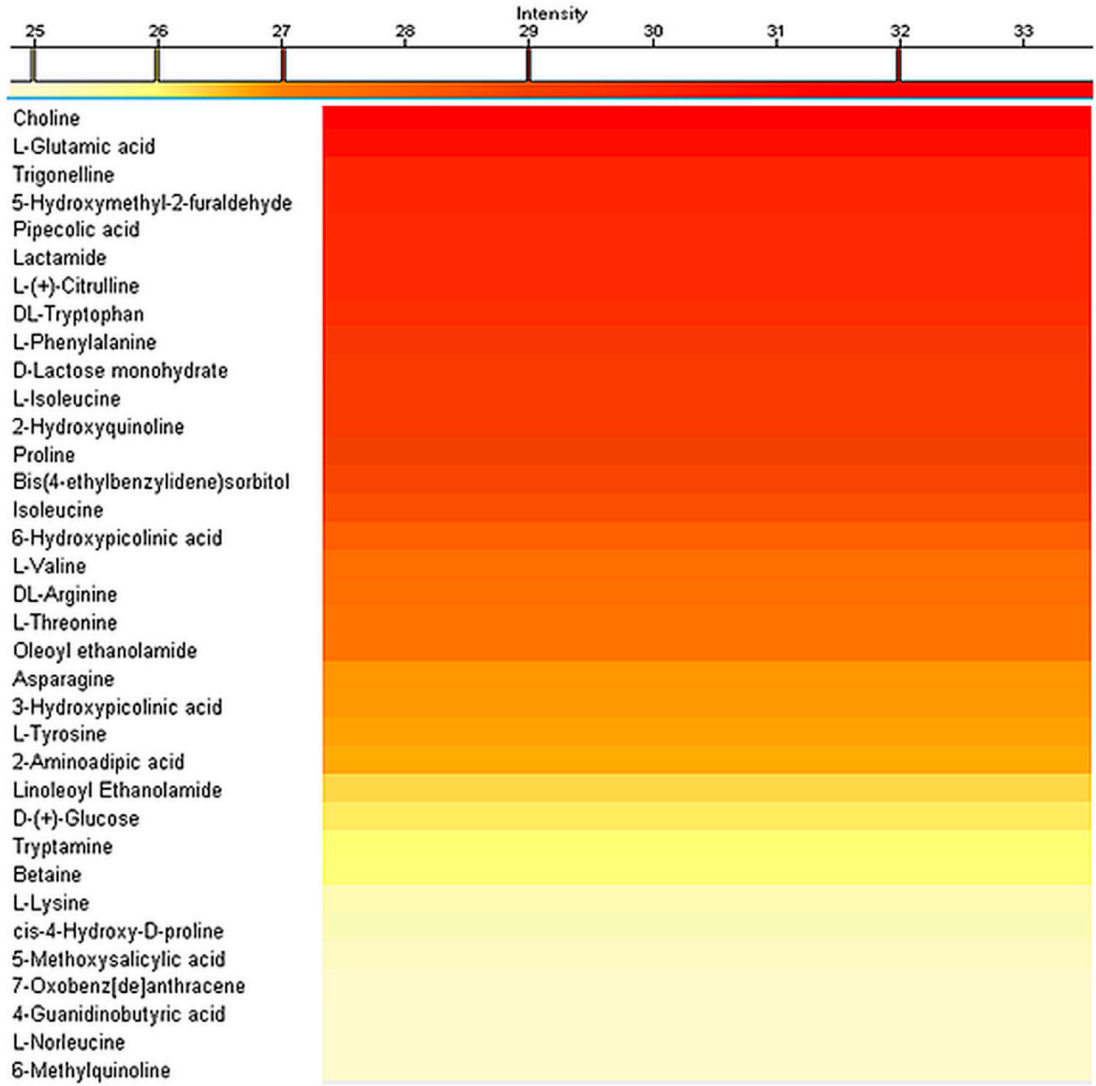
Mass spectrometry analysis of the hazelnut extracts showing the relative amount of the compounds detected by using a C18 column in positive ion mode.

The extraction concentration at the end of a typical procedure resulted to be 6.0 ± 0.5 mg/ml. Qualitative characterization of the extract has been performed by mass spectroscopy analysis to highlight the main present compounds, by using a C18 column in positive ion mode. The heat map reported in Figure 1 shows the identified metabolites and their relative quantities in the extract. Choline, the compound present at the highest amount in the extract, plays an important role in the structure of the cells, particularly in the development of membrane phospholipids; moreover, it is required in the central nervous system as a progenitor of the neurotransmitter acetylcholine. Although it is considered a non-essential nutrient, in fact, observations of people deprived of dietary choline indicate the development of dysfunctions, especially fatty liver disease and muscle damage (Zeisel and Costa, 2009). These conditions were reversed in the case of dietary choline higher intake. L-glutamic acid, the compound present in the second-highest amount in the extract, is a major excitatory transmitter in the central nervous system; moreover, it is a precursor of other neurotransmitters, in particular GABA (Garattini, 2000). Trigonelline, the third highest-amount present molecule, is a metabolite that contributes to decrease the level of glucose and lipids in the blood, besides owning neuroprotective and anti-bacterial effects (Zhou et al., 2012). In addition to the above-mentioned compounds, many other biologically active molecules, including amino acids, vitamins, and metabolites, were also identified in the extract, presenting interesting properties leading to antioxidant, antibiotic, and anti-inflammatory effects.

Having considered the mass spectroscopy results, choline content in the extract has been selected as a quantification marker to analyze the total extract quantity: at this aim, exact choline concentration was determined by using a choline assay, and results indicate the presence of 3.8 ± 0.4 µg of choline *per* 6 mg of extract. Evaluation of the total phenolic group content, performed through the Folin Ciocalteu’s test, provided that the equivalent phenolic content of 246.4 ± 12.8 µg of tannic acidic is present in 6 mg of hazelnut extract. Eventually, the total antioxidant capacity evaluation highlighted the presence of 152.6 ± 8.3 ng of Trolox equivalent in 6 mg of hazelnut extract. As already shown in the literature, a correlation between phenolic content and antioxidant activity (Piluzza and Bullitta, 2011) has been found, supposing that part of the antioxidant activity is ascribable to the presence of phenolic compounds; this notwithstanding, we cannot exclude a contribution from vitamins and fatty acids, the presence of which has been detected through the mass spectroscopy analysis.

### 3.2 Preparation and characterization of NLCs and NE_NLCs

**FIGURE 2:**
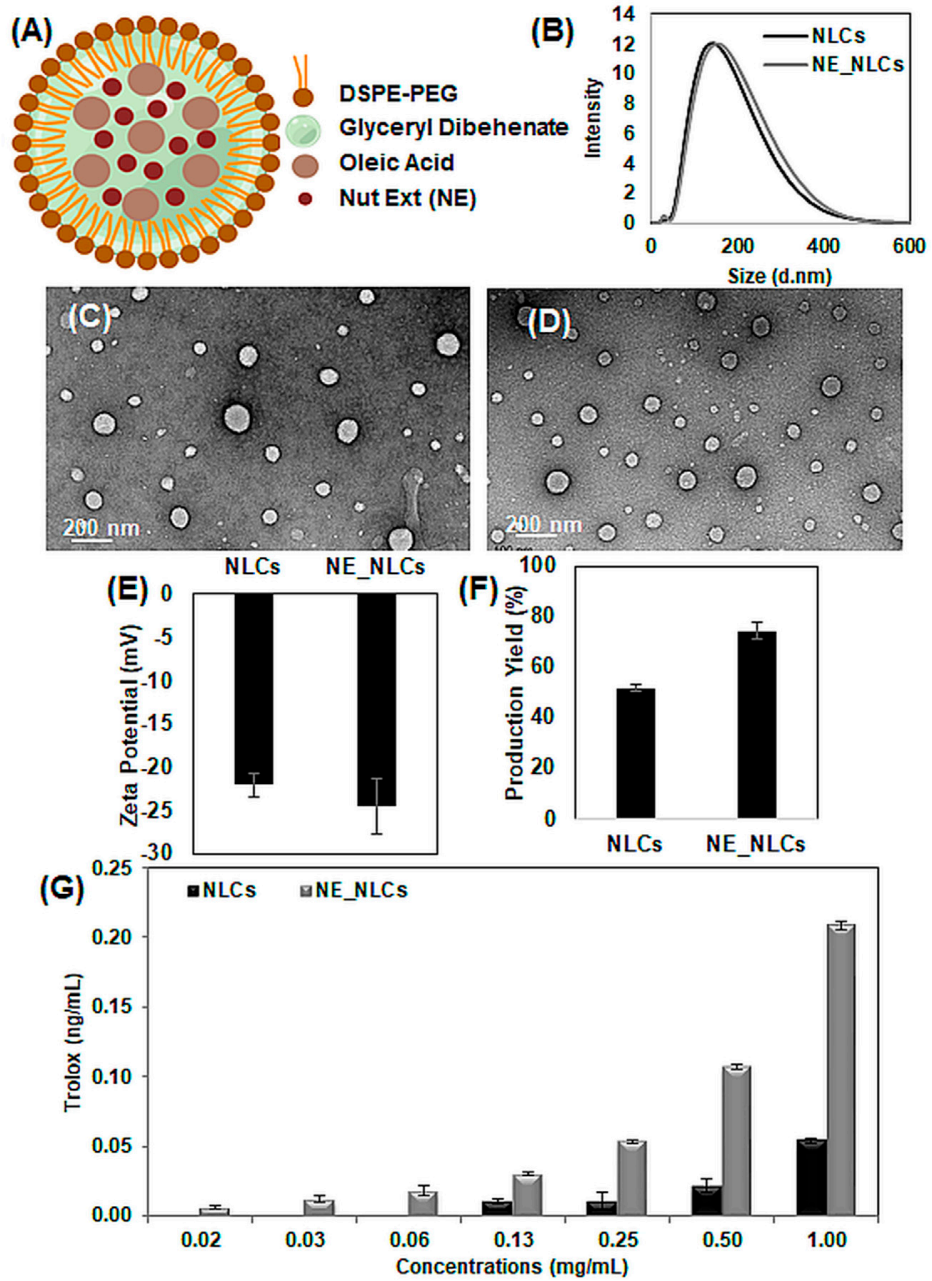
Characterization of NLCs and NE_NLCs. Schematic representation of the structure of NE_NLCs **(A)**, dynamic light scattering **(B)**, representative transmission electron microscopy (TEM) images of NLCs **(C)** and NE_NLCs **(D)**, ζ-Potential measurements **(E)**, and production yield **(F)**. Total antioxidant capacity of NLCs and NE_NLCs at increasing concentrations expressed as Trolox equivalent **(G)**.

In order to promote the stabilization in the physiological environment and the protection of the active ingredients for bio-applications, we decided to encapsulate the extracts in lipid-based nanocarriers, and namely NLCs. The loading in nanoparticles indeed generally allows for an improved biodistribution and, with an appropriate surface functionalization, enables a selective targeting of the active cargo. In Figure 2A, a schema of the resulting NE_NLCs is provided, composed of glyceryl dibehenate and oleic acid as solid and liquid lipids, respectively, as well as DSPE-PEG as a stabilizer. The hydrodynamic size distribution of NLCs and NE_NLCs measured by dynamic light scattering resulted in 142.4 ± 0.3 and 164.2 ± 1.2 nm, respectively, as shown in Figure 2B. The slight increment in the size of the NE_NLCs with respect to the bare NLCs is probably due to steric modification provided by the entrapped extract. Concerning the morphological characterization, representative TEM images of NLCs and NE_NLCs are reported in Figures 2C,D, respectively, and show the highly uniform and spherical structure of the nanovectors. The uniform size distribution is moreover confirmed by the polydispersity index (PDI), in both cases <0.4. The colloidal stability of the nanovectors, evaluated through ζ-potential assessment, is excellent, as demonstrated by values of -22.1 ± 1.4 and -24.5 ± 3.2 mV for NLCs and NE_NLCs, respectively (Figure 2E); being ζ-potential below -20 mV, satisfactory stability in the physiological environment is expected. The production yield of the NLCs and NE_NLCs resulted to be 51.6 ± 1.4% and 74.3 ± 3.4%, respectively (Figure 2F). The significant increment in the production yield of the NE_NLCs formulation is supposedly attributed to the presence of the extract that could further stabilize the nanoparticle components during their synthesis. The encapsulation efficiency, indirectly assessed through the determination of the choline concentration, resulted to be 70%, while a release behavior analogous to similar nanosystems is expected (Martinelli et al., 2020; Sen et al., 2021). Finally, the total antioxidant capacity of the nanovectors (both plain NLCs and NE_NLCs; 0.02, 0.03, 0.06, 0.13, 0.25, 0.50, and 1.00 mg/ml) was evaluated in terms of Trolox equivalents (Figure 2G). A linear correlation between nanovector concentration and antioxidant power was found, as expected, as well as a significantly higher antioxidant capacity of the extract-loaded nanovectors with respect to the empty ones. In particular, 1 mg/ml of NE_NLCs correspond to 0.208 ± 0.003 ng/ml of Trolox equivalent, while 1 mg/ml of NLCs to 0.054 ± 0.002 ng/ml of Trolox equivalent antioxidant capacity. The mild antioxidant activity of the bare NLCs is most probably due to the presence of oleic acid and glyceryl dibehenate, as already highlighted in the literature (Rohmah et al., 2022).

### 3.3 Cytocompatibility of NLCs and NE_NLCs

**FIGURE 3:**
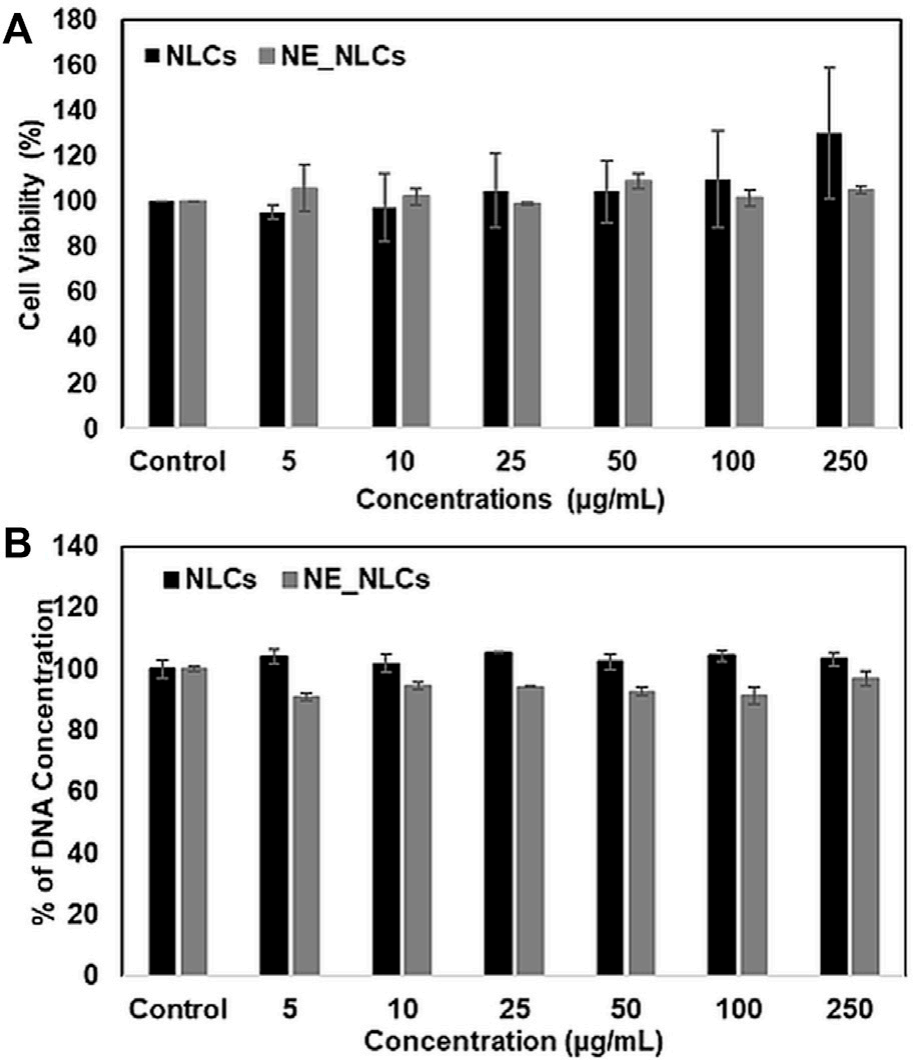
Cytocompatibility evaluation of NLCs and NE_NLCs at increasing concentrations: WST-1 metabolic assay, indicative of cell viability **(A)**, and PicoGreen dsDNA quantification, indicative of cellular proliferation **(B).** Data are represented as mean values ±standard deviation (*n* = 6).

The cytocompatibility of NLCs and NE_NLCs has been evaluated on HDFs at varying concentrations (0, 5, 10, 25, 50, 100, and 250 μg/ml) by performing WST-1 colorimetric assay to measure the metabolic activity and Pico Green dsDNA assay to analyze cell viability after 24 h of treatment (Figures 3A,B). No significant effects of the NLCs and NE_NLCs were found on both cell metabolism and proliferation, at all tested concentrations. According to the collected data, a precautionary concentration of 100 μg/mL has been selected for further experiments.

### 3.4 Cellular internalization of NE_NLCs

**FIGURE 4:**
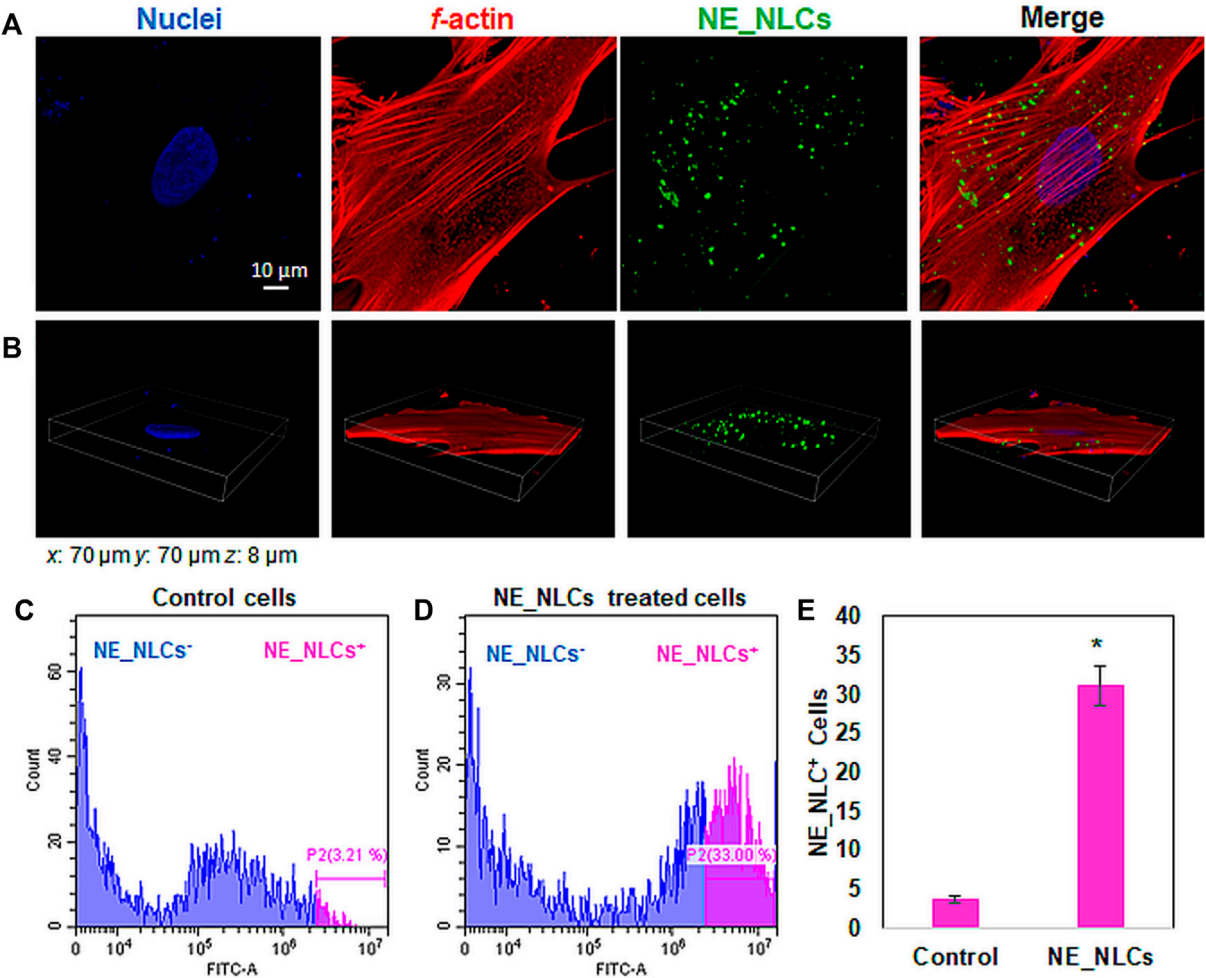
Representative confocal images of HDFs treated for 24 h with NE_NLCs 100 μg/ml (NE_NLCs in green, f-actin in red, and nuclei in blue): single *z*-stack **(A)** and 3D rendering **(B)** images. Representative flow cytometry results of control **(C)** and NE_NLCs-treated **(D)** HDFs, as well as quantitative analysis **(E)**. Data are represented as mean values ±standard deviation (*n* = 3; *p < 0.05).

The cellular localization of NE_NLCs was investigated using both confocal laser scanning microscopy imaging and flow cytometry measurement after 24 h of incubation. Figure 4 demonstrates an efficient internalization of NE_NLCs by HDFs. Confocal images of DiO-labeled NE_NLCs (in green), *f*-actin (in red), and nuclei (in blue) indicate the localization of the particles in the cytoplasm, as depicted in a representative single *z*-stack (Figure 4A), and by the 3D rendering (Figure 4B). The quantitative evaluation of the NE_NLC internalization was instead performed by flow cytometry, and the results are reported in Figures 4C–E. After 24 h of treatment, a relatively high extent of interaction was found (31.2 ± 1.9% of NE-NLCs^+^ cells). Obtained results are in line with previous studies by both our (30–37% NLC^+^ endothelial cells; Battaglini et al., 2019) and other groups (Caco-2 cells; Li et al., 2016). In general, the literature supports a time-dependent NLC uptake, that are eventually localized in the perinuclear area of the cytoplasm.

### 3.5 ROS detection

**FIGURE 5:**
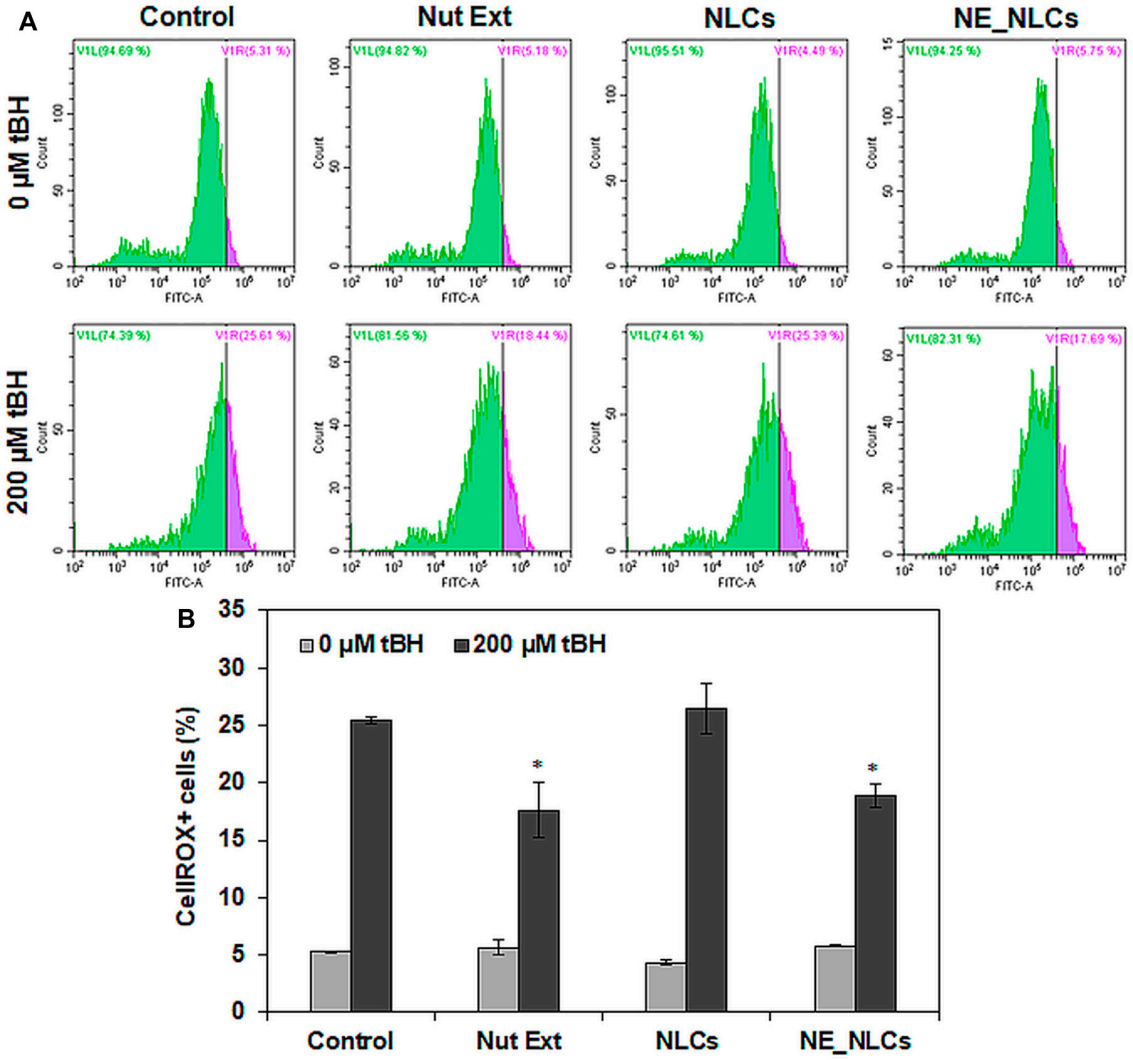
Levels of reactive oxygen species (ROS) in HDFs after 24 h of the indicated treatments (NE_NCLs 100 μg/ml, Nut Ext 70 μg/ml): representative scatter plots **(A)** and quantitative analysis **(B)**. Data are represented as mean values ±standard deviation (*n* = 3; *p < 0.05).

To evaluate the antioxidant activity of NE_NLCs, HDF cultures have been treated with a pro-oxidant insult (tBH). In the presence of tBH, untreated control cultures showed 25.4 ± 0.3% of ROS^+^ cells, while a significant decrement was observed in tBH + NE_NLCs-treated cultures (18.8 ± 1.1%, *p* < 0.05). Plain extracts were also able to induce a significant decrement of oxidative stress (17.6 ± 2.4%, *p* < 0.05), while no effects were highlighted in the case of treatment with unloaded NLCs (Figure 5).

According to the literature, hazelnuts show good antioxidant effects by downregulating lactate dehydrogenase and malondialdehyde, upregulating the activity of antioxidant enzymes (total superoxide dismutase glutathione peroxidase, and catalase), and scavenging ROS in human umbilical vein endothelial cells (HUVECs) (Fang et al., 2019). An interesting study on 24 healthy volunteers who daily consumed 40 g of hazelnuts shows modulation of oxidative stress and inflammatory genes *via* upregulating superoxide dismutase 1 (*SOD1*), catalase (*CAT*), macrophage migration inhibitory factor (*MIF*), peroxisome proliferator-activated receptor gamma (*PPARγ*), vitamin D receptor (*VDR*), methylenetetrahydrofolate reductase (*MTHFR*), and angiotensin I-converting enzyme (*ACE*) (Di Renzo et al., 2019). In another study, the reduction of ROS levels in a mouse model was attributed to coline supplementation by inhibiting the inflammatory response and modulating the redox status of bronchoalveolar cells (Mehta et al., 2009). All these data corroborate our findings and suggest hazelnut extracts as a powerful antioxidant agent suitable for medical and nutraceutical applications.

## 4 Conclusion

The hazelnut extracts include a great and diverse variety of active ingredients, in particular in terms of antioxidant compounds, especially phenols and choline. In order to promote their exploitation in nanomedicine, encapsulation in lipid-based nanocarriers has been proposed. Obtained findings highlighted the successful preparation of NE_NLCs, their optimal features in terms of stability and cytocompatibility, and efficient ROS scavenging effects on HDFs. Altogether, our results encourage further investigations, also envisioning biomolecular analyses and *in vivo* testing, and applications of hazelnut extracts-based nanomedical products.

## Data Availability

The raw data supporting the conclusions of this article will be made available by the authors, without undue reservation.
